# Study on the Performance and Service Life Prediction of Corrosion-Resistant Concrete Cut-Corner Square Piles

**DOI:** 10.3390/ma18163776

**Published:** 2025-08-12

**Authors:** Rui Sheng, Kang Wang, Hua Wei, Hao Lu, Chunhe Li

**Affiliations:** 1 College of Civil Engineering, Nanjing Tech University, Puzhu South Road No. 30, Nanjing 211816, China; sr-@njtech.edu.cn; 2 Materials & Structural Engineering Department, Nanjing Hydraulic Research Institutes, Nanjing 210029, China; 18964261589@163.com (K.W.); hwei@nhri.cn (H.W.); 3 College of Water Conservancy and Hydropower Engineering, Hohai University, Nanjing 210098, China; 4 Civil and Environmental Engineering, The University of Miyazaki, 1-1 Gakuenkibanadainishi, Miyazaki 889-2192, Japan

**Keywords:** concrete pile foundation, chloride ion corrosion, finite element simulation, service life prediction

## Abstract

This paper addresses the issue of reduced lifespan of coastal concrete piles due to chloride ion corrosion. A combination of concrete mix optimization and pile geometry improvement measures is proposed. Based on the diffusion coefficient optimization of Fick’s second law, the service life prediction of concrete piles in corrosive environments is completed. The results show that, compared to single slag incorporation and the “slag-fly ash” dual-component mix, the “slag-fly ash-corrosion inhibitor” triple-component concrete achieves a 28-day compressive strength of 67.4 MPa, and the chloride ion diffusion coefficient is reduced to 1.14 × 10^−12^ m^2^/s, significantly improving overall performance. Finite element simulations reveal that, compared to ordinary square piles, cut-corner square piles can effectively alleviate stress concentration at the pile tip and reduce settlement. The maximum stress is 3.94 MPa, and the settlement is 22.64 mm, representing reductions of about 16.3% and 15.5%, respectively, compared to ordinary square piles. Concrete service life prediction confirms that the concrete with corrosion inhibitors has a predicted service life of 31.5 years, extending 7.4 years and 13.3 years longer than the single slag and the “slag-fly ash” dual-component groups, respectively. The “material-structure” optimization theory proposed in this study provides a theoretical basis and technical path for the long-life design of coastal engineering pile foundations.

## 1. Introduction

Coastal and offshore engineering projects are generally more severely affected by erosion and damage to concrete piles due to the physical and chemical actions of seawater and salt fog, compared to inland structures. The chemical effects mainly include the corrosion of concrete by corrosive agents such as sulfate, magnesium salts, and chloride salts in seawater. The physical effects include salt crystallization pressure caused by repeated wetting and drying cycles [1,2,3,4]. According to surveys, the economic losses caused by corrosion in China amount to at least CNY 40 billion annually [5].

Chloride ion-induced corrosion is the main cause of the reduced lifespan of structures in marine wet/dry cycling areas. To address this issue, improvements can be made in both the corrosion resistance of concrete pile materials and the optimization of pile geometry [6]. In terms of materials, to enhance the durability of concrete piles in coastal areas, Ding Jiantong [7] replaced ordinary cement with sulfate-resistant cement. The results showed that sulfate-resistant cement performed better in resisting corrosion from chloride and sulfate solutions compared to ordinary Portland cement. Guang-Zhu Zhang [8] explored the effect of modified basalt fibers, with nano-SiO_2_ as a modifier and expanded perlite as a carrier for self-healing agents, on the self-healing properties of microbial mortar and the interface compatibility, thus explaining the improvement in durability. Rongzhen Piao [9] found that adding nano-SiO_2_ significantly increased the packing density of ultra-high-performance concrete, producing rich calcium (aluminum) silicate hydrate gels, which improved the density and reduced the overall porosity, leading to noticeable improvements in durability. Sihong He [10] considered factors such as the chloride ion diffusion coefficient, permeability coefficient, reverse osmosis pressure, initial crack characteristics, and centrifugal layering, analyzing the effect of saturated active protection technology (RS-AAD) on the convective inhibition of chloride ion diffusion. Hossack and Siad et al. [11,12] analyzed chloride ion corrosion at different surface depths of concrete under various wet/dry cycles. With the increase in wet/dry cycles, the demineralization of C-S-H gel led to a reduction in the number of well-crystallized C-S-H network gels, causing the concrete’s compactness to decrease, thus accelerating the migration of chlorides within the matrix. In addition to material improvements, optimizing the geometric structure of concrete piles to reduce the settlement depth under pile loads can effectively reduce chloride ion penetration. Luo Xiaoyong [13] established a practical constitutive model for concrete subjected to hydrochloric acid corrosion under repeated loading based on the optimized effective bearing cross-sectional area ratio, and validated the model. The research results greatly enrich the protective structural measures for aging buildings in coastal areas. In recent years, with the deepening research on the durability of concrete under corrosive environments, numerous scholars have adopted constitutive modeling and numerical simulation approaches to investigate the effects of degradation mechanisms on the performance of concrete structures. Among them, continuum damage mechanics (CDM) effectively describes corrosion-induced stiffness degradation and microcrack evolution by introducing damage variables into constitutive relationships [14]. In contrast, fracture mechanics-based methods are more suitable for simulating crack propagation and failure under chloride-induced corrosion [15]. For instance, Qiu [16] developed a multi-field damage constitutive model incorporating chloride diffusion coupling, which accurately predicts the degradation behavior of concrete during service life. Korec [17] proposed a phase-field–chloride-coupled evolution model to reveal the crack-driving mechanism of concrete under non-steady chloride diffusion processes. However, such geometric optimization methods still have certain limitations. For instance, although structural improvements can suppress stress concentration at the pile tip, they have limited effectiveness in hindering the diffusion of chloride ions deep into the pile [18]. By optimizing the concrete pile structure to minimize vertical settlement during loading, it is possible to reduce long-term exposure to seawater, thereby improving the service life of the pile [19,20].

**Figure 1:**
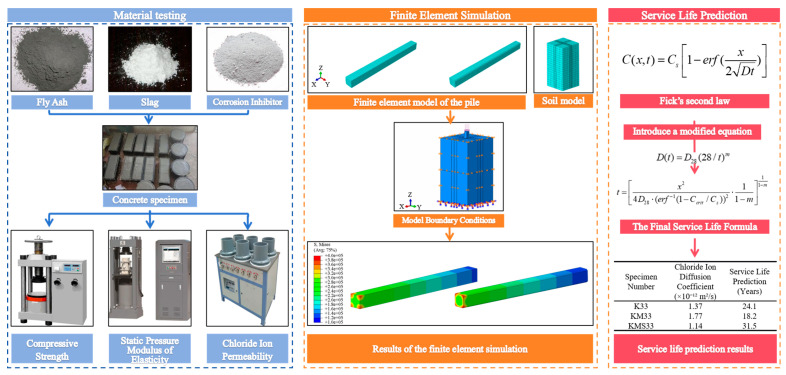
Research Framework.

This study is conducted based on a dual-optimization strategy involving both material and structural levels. At the material level, high-performance concrete (HPC) is adopted as the base matrix, with the incorporation of mineral admixtures and corrosion inhibitors to enhance both mechanical strength and long-term durability. Compared with conventional concrete, HPC exhibits superior workability, higher compressive strength, and significantly improved resistance to chloride ion penetration. These advantages stem from its lower water-to-binder ratio, refined pore structure, and the synergistic effects of supplementary cementitious materials and chemical admixtures, which collectively reduce permeability and mitigate the ingress of aggressive ions. Such characteristics make HPC particularly well suited for marine pile foundations, where structures are continuously exposed to chloride-rich environments and long-term durability is critical. At the structural level, a finite element model of a cut-corner square pile is designed to alleviate stress concentration at the pile tip, thereby improving load transfer efficiency and enhancing overall structural synergy. Furthermore, a modified Fick’s second law model is applied to quantitatively evaluate the service life of different concrete mixtures under marine exposure, based on the microscopic chloride diffusion mechanism. Together, these strategies provide a theoretical foundation and technical pathway for the long-term performance design of pile structures. The overall research framework is illustrated in Figure 1.

## 2. Material Testing Study

### 2.1. Raw Materials

#### 2.1.1. Powders

The powders used in the experiment primarily include cement, fly ash, and slag. The cement used is Union brand P·O 52.5 ordinary Portland cement. The chemical composition of each powder was tested, and the results are shown in Table 1.

#### 2.1.2. Aggregates

The fine aggregate used is river sand, and the coarse aggregate is artificial crushed stone with a continuous gradation. The particle size of the crushed stone ranges from 5 mm to 31.5 mm. The physical properties of the aggregates are shown in Table 2.

#### 2.1.3. Admixtures

The admixtures used include a fat-based high-efficiency water-reducing agent produced by Xuzhou Julong Admixture Co., Ltd. (Xuzhou, China) and an air-entraining agent HK-F2 produced by Nanjing Ruidi High-Tech Co., Ltd. (Nanjing, China). The admixtures were tested according to the standards in “Concrete Admixtures” (GB 8076-2008) [21], and the test results are shown in Table 3. The performance test results indicate that both the fat-based high-efficiency water-reducing agent and the air-entraining agent HK-F2 meet the required standards.

#### 2.1.4. Corrosion Inhibitor

The corrosion inhibitor was developed by the Nanjing Hydraulic Research Institute. It is designed for environments with medium to strong corrosion from chloride salts and sulfate salts. This product enhances the durability and volume stability of concrete. The quality specifications of the corrosion inhibitor are shown in Table 4.

### 2.2. Experimental Methods

#### 2.2.1. Mix Design

In this study, three types of corrosion-resistant concrete mix ratios were designed. The water-to-binder ratio was kept constant at 0.33. The reference group, K33, used a slag/cement binary system, with slag micro-powder enhancing pore refinement and improving impermeability. The optimized group, KM33, introduced 10% fly ash to form a ternary composite binder system, utilizing the spherical particle effect to improve workability and collaborate in densifying the microstructure. The corrosion-resistant group, KMS33, innovatively adopted a quaternary cementitious system, where the calcium aluminate generated by the corrosion inhibitor and the chloride ion anchoring mechanism enhance the chemical curing ability. The mix design is shown in Table 5.

All raw materials used in specimen preparation, including cement, slag, fly ash, corrosion inhibitor, sand, and coarse aggregates, were precisely weighed using a calibrated electronic balance with an accuracy of ±0.1 g. Mixing was performed using a JS500 forced mixer (Zhengzhou Jinhuiyuan Machinery Manufacturing Co., Ltd., Zhengzhou, China). Initially, the dried cementitious materials, fine aggregates, and coarse aggregates were dry-mixed for 60 s. Then, water premixed with admixtures was gradually added, followed by continuous mixing for an additional 120 s. The corrosion inhibitor was pre-dissolved in the mixing water to ensure uniform dispersion. The mixing procedure followed the standard method specified in GB/T 50081-2019: Standard for Test Method of Mechanical Properties on Ordinary Concrete [22]. After mixing, the fresh concrete was poured into standard molds, compacted, and formed. The specimens were then cured under standard curing conditions (temperature: 20 ± 2 °C, relative humidity: ≥95%) until the designated testing age.

#### 2.2.2. Compressive Strength

According to the GB/T 50081-2019 standard [22], the concrete compressive strength test uses standard cubic specimens with dimensions of 150 mm × 150 mm × 150 mm. The specimens are loaded axially on a pressure testing machine at a loading rate of 0.5 MPa/s until failure occurs. The final strength value is represented by the arithmetic mean of the compressive strengths of three parallel specimens.

#### 2.2.3. Static Pressure Modulus of Elasticity

In accordance with GB/T 50081-2019 [22], the concrete static compressive modulus of elasticity test uses prismatic specimens with dimensions of 150 mm × 150 mm × 300 mm. During the test, an initial load of 0.5 MPa is applied at a rate of 0.5 MPa/s, and the specimen is preloaded three times to eliminate contact gaps. The specimen is then loaded to the stress level corresponding to one-third of the axial compressive strength and held for 60 s. The elastic deformation values are measured on both sides using symmetrically arranged strain gauges.

#### 2.2.4. Chloride Ion Penetration

According to the GB/T 50082-2024 standard [23], the concrete chloride ion permeability test uses specimens with dimensions of Φ100 mm × 50 mm. The test is conducted using the Rapid Chloride Migration (RCM) method and the Electric Flux Method. The test results are determined by the average of three sets of results. The tests were conducted under controlled environmental conditions with a temperature of 20 ± 2 °C and a relative humidity of 60% ± 5%. Prior to testing, the specimens were fully saturated to ensure the accuracy and repeatability of the diffusion measurements. The final results were determined based on the average values of three parallel specimens.

### 2.3. Experimental Results

#### 2.3.1. Strength Characteristics

**Figure 2:**
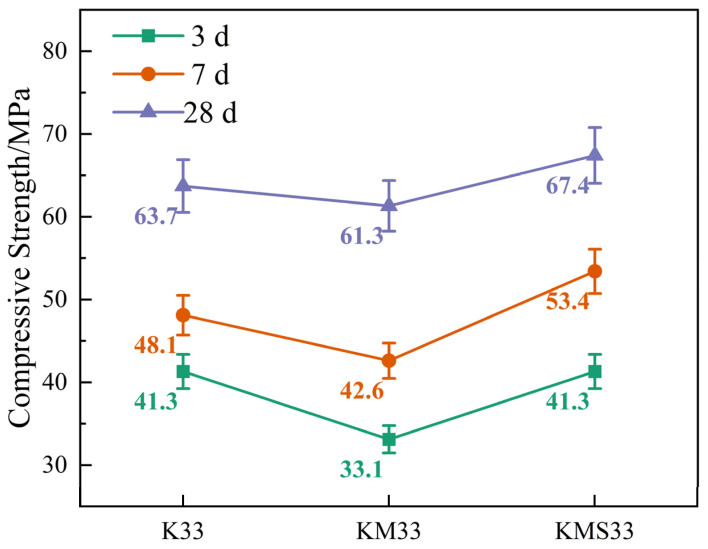
Compressive strength of concrete specimens at different ages.

The variation in compressive strength of the three groups of specimens (K33, KM33, and KMS33) at 3, 7, and 28 days is shown in Figure 2.

Overall, all specimens exhibited a significant increase in compressive strength with curing age, demonstrating a typical strength development trend of cement-based materials. Notable differences in strength performance were observed among the three groups. For the K33 group, the average compressive strength at 3 days was 41.3 MPa with a standard deviation of 2.1 MPa. In contrast, the KM33 group showed a lower early strength of 33.1 MPa with a deviation of 1.7 MPa, attributed to the slower early hydration of the binary blended system. The KMS33 group, incorporating ettringite-promoting components, enhanced the nucleation sites for cement hydration, thereby compensating for early reaction limitations. As a result, its 3-day strength reached 41.3 MPa with a deviation of 2.0 MPa, comparable to that of K33, indicating improved early strength and consistency.

At 7 and 28 days, all three groups showed further increases in compressive strength. The KMS33 group, benefiting from improved compactness due to mineral admixtures and ongoing pozzolanic reactions, reached 53.4 MPa and 67.4 MPa at 7 and 28 days, respectively, with standard deviations of 2.6 MPa and 3.4 MPa. These values represent increases of 11.0% and 5.8%, respectively, over the K33 group (48.1 MPa and 63.7 MPa). The KM33 group also exhibited significant strength development, reaching 61.3 MPa at 28 days—an 85.8% increase from its 3-day value. However, its standard deviation increased to 3.1 MPa, suggesting some degree of variability remained in the reaction process. Overall, the error bars in the figure indicate good repeatability of the compressive strength measurements for all groups. Notably, the KMS33 specimens demonstrated superior stability and homogeneity across all ages, confirming the beneficial effects of the ternary blended cementitious system in enhancing matrix densification and hydration uniformity.

#### 2.3.2. Elastic Modulus

**Figure 3:**
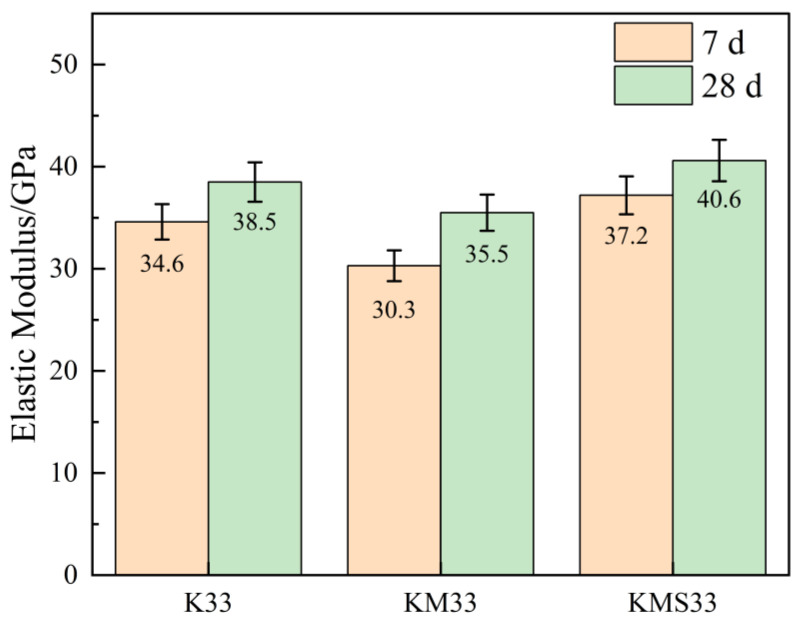
Elastic modulus of concrete specimens at different ages.

The variations in elastic modulus of concrete at 7 and 28 days for different mix designs are shown in Figure 3. The elastic modulus of all groups increased with curing age, indicating that the material structure became denser over time due to continued hydration, thereby enhancing stiffness. For the K33 group, the elastic modulus reached 34.6 GPa at 7 days and 38.5 GPa at 28 days, with standard deviations of 1.7 GPa and 1.9 GPa, respectively—an increase of 11.3%. This reflects the ongoing hydration of slag and its contribution to the stiffness development of concrete. In the KM33 group, the early-age elastic modulus was only 30.3 GPa at 7 days (standard deviation: 1.5 GPa), due to the low early reactivity of fly ash [24]. However, it increased to 35.5 GPa at 28 days (standard deviation: 1.8 GPa), corresponding to a 17.2% increase, highlighting the delayed pozzolanic effect of fly ash.

The KMS33 group, benefiting from the early nucleation and micro-filling effects of the corrosion inhibitor, along with the synergistic action of the ternary cementitious system, achieved an elastic modulus of 37.2 GPa at 7 days (standard deviation: 1.9 GPa), which is approximately 7.5% higher than that of K33. At 28 days, the modulus further increased to 40.6 GPa with a standard deviation of 2.0 GPa. These results demonstrate that the ternary blended system not only promotes early stiffness development but also significantly enhances matrix densification and the long-term growth of elastic modulus.

Overall, the short error bars across all ages indicate low variability and good repeatability of the test results. Among all groups, KMS33 exhibited the most pronounced improvement in elastic modulus and the highest data consistency.

#### 2.3.3. Chloride Ion Permeability

**Figure 4:**
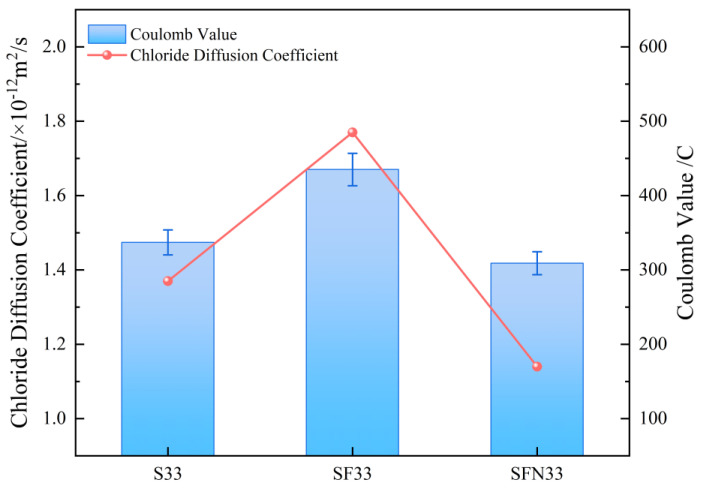
Chloride ion permeability of concrete specimens.

The electrical flux and chloride ion diffusion coefficients of the concrete specimens from each group are shown in Figure 4.

The results indicate that the KMS33 mixture exhibited the most superior resistance to chloride ion penetration, with a charge passed of only 309 C and a chloride diffusion coefficient of 1.14 × 10^−12^ m^2^/s, representing reductions of 8.3% and 16.8%, respectively, compared with the reference mixture K33. This remarkable improvement can be mainly attributed to the synergistic effects of the corrosion inhibitor: on one hand, the active components in the inhibitor promote the formation of abundant ettringite (AFt) and stable salt compounds during hydration, which chemically bind with the intruding Cl^−^ ions and markedly suppress their free migration; on the other hand, the micro-filling effect of the inhibitor, together with slag, effectively refines the pore structure, decreases the connectivity of capillary pores, and prolongs the diffusion paths of chloride ions. In contrast, the KM33 mixture exhibited the poorest performance, with a charge passed of 435 C and a diffusion coefficient of 1.77 × 10^−12^ m^2^/s, due to the low early reactivity of fly ash, which led to insufficient initial densification of the matrix. Although the pozzolanic reaction contributed at 28 days, the porosity remained relatively high. The K33 mixture showed intermediate performance, where the continuous hydration of slag partially improved the microstructure but failed to achieve a chemical binding effect comparable to that of KMS33. These findings demonstrate that KMS33 exhibits significantly enhanced durability compared with conventional mineral admixture systems.

## 3. Numerical Simulation Study

### 3.1. Model Construction

#### 3.1.1. Geometric Model

**Figure 5:**
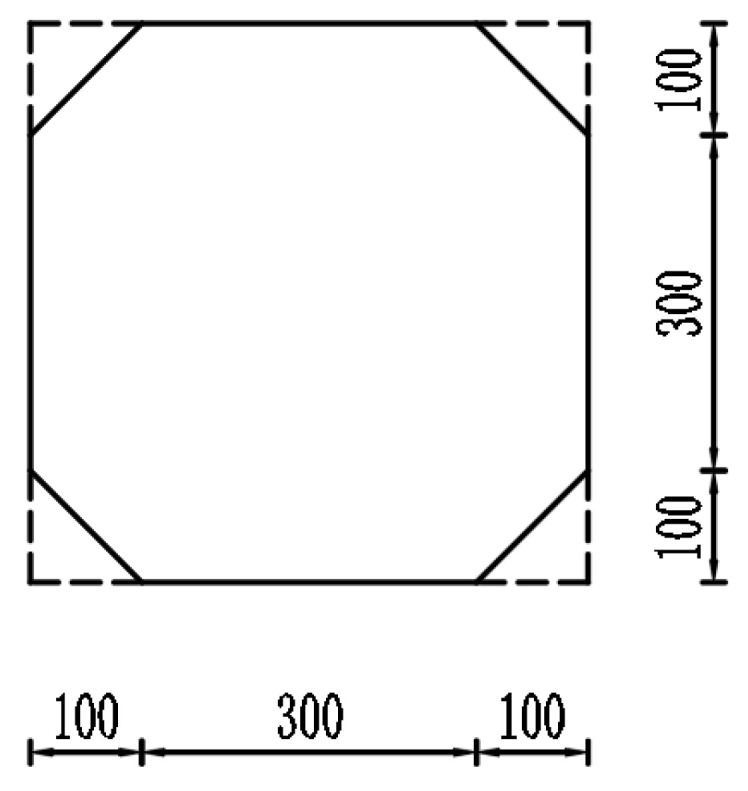
Pile section dimensions (unit: mm).

The vertical bearing performance of standard square piles and cut-corner square piles was analyzed using ABAQUS 2021 (Dassault Systèmes, Vélizy-Villacoublay, France) finite element software. The cross-sectional geometries of the two types of piles are illustrated in Figure 5. The standard square pile features a conventional square cross section with a side length of 500 mm. In contrast, the cut-corner square pile is symmetrically chamfered at all four corners, with each chamfer formed by cutting an isosceles right triangle along a 45° angle. Each chamfer has a leg length of 100 mm, resulting in an octagonal cross section. This chamfering approach aims to reduce stress concentration at the pile corners and improve the stress distribution at the pile–soil interface.

**Figure 6:**
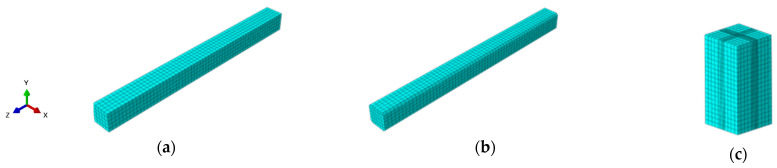
Three-dimensional finite element model of pile–soil system: (**a**) ordinary square pile, (**b**) cut-corner square pile, (**c**) soil.

The pile length is 10 m, with an embedded depth of 8 m in the soil. The surrounding soil domain measures 5 m × 5 m × 20 m. The three-dimensional finite element model of the pile–soil system is shown in Figure 6. Both the pile and the soil are discretized using C3D8R elements—eight-node linear brick elements with reduced integration. A mesh size of 100 mm is used for the pile body, while a coarser mesh of 500 mm is applied to the soil domain. Local mesh refinement is introduced in the pile–soil contact region, with the minimum element size set to 50 mm to enhance contact analysis accuracy.

The interaction between the pile and the soil is modeled using surface-to-surface contact. In the normal direction, a “hard contact” model is adopted, while in the tangential direction, frictional behavior is simulated using a penalty function method based on Coulomb’s friction law, with a friction coefficient of 0.45. This contact model effectively captures the slip and force transfer behavior at the pile–soil interface.

#### 3.1.2. Material Parameters

The corrosion-resistant concrete determined by the KMS33 mix ratio is used as the pile material (KMS). The material parameters for both the concrete and the excavation soil were calibrated through material performance tests, as shown in Table 6.

#### 3.1.3. Boundary Conditions

**Figure 7:**
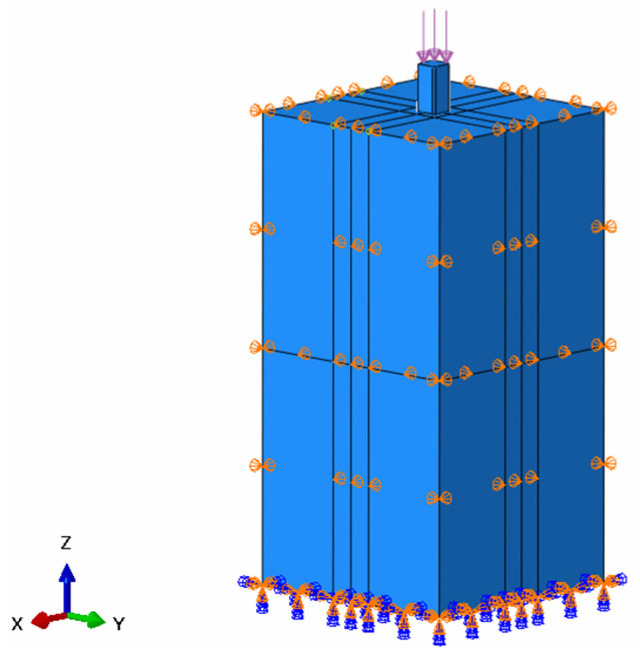
Model boundary conditions.

The boundary conditions for this model are defined as shown in Figure 6. The base is fully fixed with constraints, and normal displacement limits are applied to the surrounding surfaces. For the pile–soil contact interface behavior, the normal direction is defined as a “hard contact” mode, and the tangential direction uses an elastic slip-based friction constitutive model, following Coulomb’s friction theory. During the geostress equilibrium phase, the theoretical analytical method is used, and the initial stress field of the pile–soil system is explicitly defined using the initial conditions, type = stress, geostatic keyword in the ABAQUS platform. To eliminate contact anomalies caused by the gravity gradient, a staged density assignment strategy is applied. During the geostress equilibrium, the pile density is temporarily set to the soil density value. After equilibrium is reached, the true material density is restored in the subsequent analysis steps to accurately establish the initial contact state of the pile–soil system [25]. The boundary conditions of the overall model are shown in Figure 7.

Based on material tests, the compressive strength of the corrosion-resistant concrete is 67.4 MPa, and the ultimate compressive bearing capacity is 1516.5 kN. To study the pile’s bearing performance under vertical load, a full simulation of the pile’s loading process is conducted, applying a vertical load of 2000 kN (approximately one time the concrete’s ultimate compressive bearing capacity) to the pile top.

### 3.2. Simulation Results

**Figure 8:**
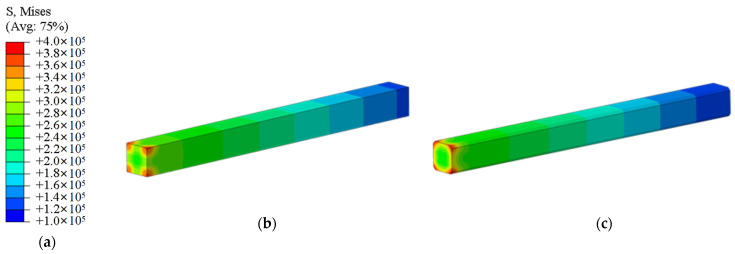
Stress distribution cloud diagram of the pile body: (**a**) legend, (**b**) ordinary square pile, (**c**) cut-corner square pile.

**Figure 9:**
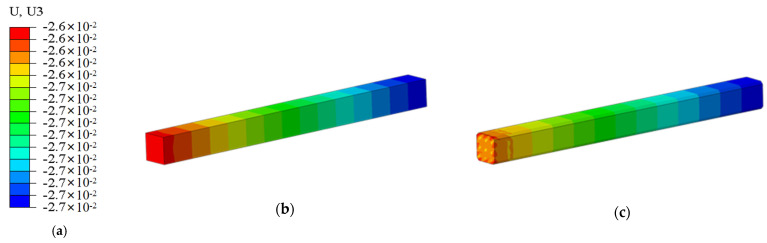
Settlement distribution cloud diagram of the pile body: (**a**) legend, (**b**) ordinary square pile, (**c**) cut-corner square pile.

The stress distribution of the pile body is shown in Figure 8, and the comparison of stress distribution between ordinary square piles and cut-corner square piles is shown in Figure 9. The results indicate that both ordinary square piles and cut-corner square piles experience stress concentration at the cut corners at the pile bottom. In the case of the ordinary square pile, the vertical stress increases significantly with depth, reaching a maximum stress of 4.87 MPa, with a sudden stress change near the bottom. In contrast, the cut-corner square pile, through its chamfered design, effectively alleviates local stress concentration, with a maximum stress of 3.94 MPa, which is approximately 16.3% lower than that of the ordinary square pile.

The settlement distribution of the pile body is shown in Figure 9. The maximum settlement of the cut-corner square pile is 22.64 mm, which is 15.5% less than the settlement of the ordinary square pile (26.80 mm). This shows that geometric optimization not only reduces stress concentration at the pile tip but also reduces the cumulative plastic deformation of the soil by improving the stress distribution at the pile–soil interface. The cut-corner design suppresses the “wedge effect” at the pile bottom and improves the pile’s stiffness continuity. This helps to transfer the load-bearing resistance from the four corners at the pile tip to the side edges at the pile bottom, turning point loads into linear loads. As a result, the local stress peak is reduced, while the overall load-bearing coordination is enhanced.

**Figure 10:**
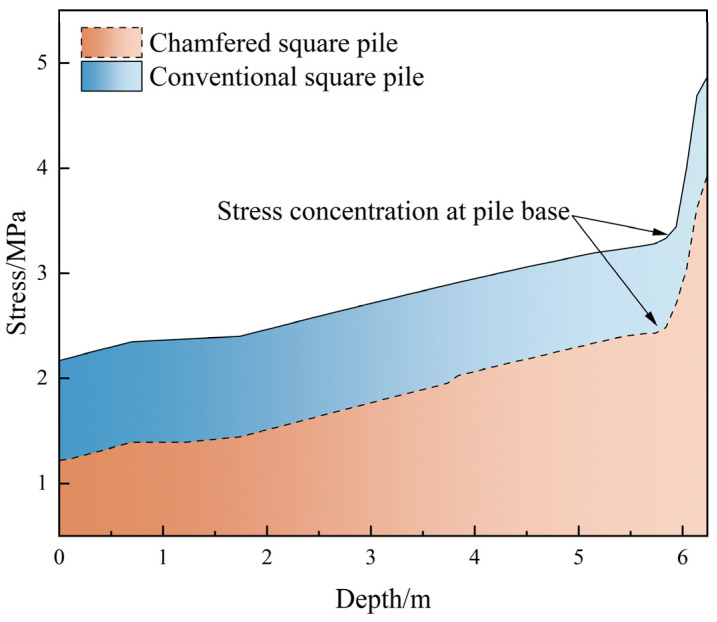
Comparison of pile body stress distribution.

Further stress distribution comparisons are presented in Figure 10. It can be observed that the stress in both pile types increases progressively with depth, with notable concentration occurring near the pile base. However, the chamfered square pile effectively reduced the magnitude of stress concentration compared with the conventional square pile, demonstrating a more uniform stress distribution along the pile depth. This finding further confirms the structural advantage of chamfered geometry under vertical loading, in which mitigating pile–base stress concentration and controlling soil plastic deformation act synergistically to improve the overall stability and reliability of the pile.

## 4. Service Life Prediction of Concrete in Corrosive Environments

### 4.1. Service Life Prediction Model

The service life of piles in corrosive environments is an important factor for their construction and application. This study builds a service life prediction model for corrosion-resistant concrete cut-corner square piles based on Fick’s second law, incorporating the time-dependent change in the diffusion coefficient into existing empirical parameters. The durability of the pile is then validated according to the actual thickness of the protective layer on the square pile.

Since the early 1970s, Fick’s second law has been widely used to calculate the depth of chloride ion penetration in concrete and predict the onset of rebar corrosion. The calculation model is as follows:

(1) $$C(x,t)=C_{s}\left[1-erf(\frac{x}{2\sqrt{Dt}})\right]$$

In the equation, *C*(*x*,*t*) represents the chloride ion content at depth *x* after time *t* in the concrete. For service life prediction, a conservative approach is taken, where the critical chloride ion concentration for marine concrete splash zones is referred to as a benchmark. *C*_s_ represents the chloride ion content at the concrete surface, *D* is the diffusion coefficient of chloride ions in the concrete, and *t* is the design service life.

When the critical chloride ion concentration is reached, i.e., *C*(*x*,*t*) = *C_crit_*, and considering the time-dependent decay of the diffusion coefficient, the modified Fick’s second law equation is as follows:

(2) $$D(t)=D_{28}{\left(28/t\right)}^{m}$$

In the equation, *D*_28_ represents the chloride ion diffusion coefficient at 28 days, and *m* is the aging coefficient, in accordance with the Code for Quality Control of High Performance Concrete in Port Engineering (JTS 257-2-2012) [26] taken as 0.2.

By substituting variables and using an approximate analytical solution, the final service life formula is as follows:

(3) $$t={\left[\frac{x^{2}}{4D_{28}\cdot {\left(erf^{-1}(1-C_{crit}/C_{s})\right)}^{2}}\cdot \frac{1}{1-m}\right]}^{\frac{1}{1-m}}$$

### 4.2. Service Life Prediction and Validation

According to the “Durability Design Code for Concrete Structures” (GB/T 50476-2019) [27], this study assumes a concrete cover thickness of 55 mm. The chloride ion content at the concrete surface, Cs, is referenced from the Ministry of Transport of China’s “Quality Control Standards for High-Performance Concrete in Harbor Engineering” (JTS257-2-2012) [26], with a value of 0.9% for the most severe environment. Referring to the same standard [26], the critical chloride ion concentration, *C_crit_*, for northern China is taken as 0.06% (by concrete mass percentage). The diffusion coefficient, *D*, decreases over time due to the effect of environmental chloride ions and follows an exponential decay law. This study adopts a value of 0.5, based on the same standard [26].

Using Equation (3) as the basic model, the service life of reinforced concrete can be calculated based on the chloride ion diffusion coefficient obtained from material tests. With a 55 mm protective layer thickness, the service life of the reinforced concrete is computed. The results are detailed in Table 7.

The service life prediction results are presented in Table 7. The predicted service lives of K33, KM33, and KMS33 were 24.1, 18.2, and 31.5 years, respectively. The comparison clearly indicates that variations in concrete composition exert a decisive influence on structural durability. Among the three mixtures, KMS33 demonstrated the best performance, extending service life by 7.4 years compared with K33 and by 13.3 years compared with KM33. This significant improvement can be attributed not only to the reduction in the chloride diffusion coefficient but also to the combined effects of the corrosion inhibitor. During hydration, the inhibitor promotes the formation of ettringite and calcium chloroaluminate, which chemically bind with penetrating chloride ions, thereby substantially slowing their free migration within the pore solution. In addition, the synergistic micro-filling effect of the inhibitor and slag effectively refines the capillary pore structure, reduces pore connectivity, and further prolongs the time required for chloride ions to reach the reinforcement.

Moreover, the finite element simulation results revealed that the geometric optimization of the cut-corner square pile played a crucial structural role. By mitigating stress concentrations at the pile toe, this design markedly reduced local stress peaks and settlement deformation, thereby lowering the risk of microcrack initiation and propagation. This structural effect not only improved load-bearing capacity but also minimized the likelihood of accelerated ingress of corrosive agents through cracks. The combined enhancement of material resistance and structural optimization thus produced a synergistic effect, enabling KMS33 to achieve superior durability compared with either material modification or geometric improvement alone.

Overall, KMS33 exhibited a predicted service life exceeding 30 years. This finding demonstrates that it can meet structural safety and durability requirements while substantially reducing maintenance and strengthening needs, thereby extending the full life cycle of the pile foundation. Such performance not only improves the overall cost-effectiveness and reliability of the structure but also provides robust technical support for the design of concrete piles in aggressive coastal environments.

## 5. Conclusions

This study focused on the service life of reinforced concrete piles in marine corrosive environments, integrating experimental tests on corrosion-resistant concrete materials, finite element numerical simulations, and service life predictions. Based on the dual-optimization concept of “material–structure”, the following main conclusions and insights were obtained:

1. Systematic material performance tests demonstrated that the ternary blend of slag–fly ash–corrosion inhibitor concrete exhibited superior mechanical and durability properties. At 28 days, the compressive strength reached 67.4 MPa, the elastic modulus was 40.6 GPa, and the chloride diffusion coefficient was reduced to 1.14 × 10^−12^ m^2^/s. The improvement can be attributed to the incorporation of corrosion inhibitors, which promoted the generation of hydration products and refined the pore structure, thereby reducing porosity and effectively blocking chloride migration pathways.

2. The finite element simulation results indicated that cut-corner square piles significantly mitigated local stress concentration at the pile tip compared with ordinary square piles. The maximum stress decreased by approximately 16.3%, and the vertical settlement was reduced by 15.5%. Such geometric optimization not only improved the stress distribution along the pile–soil interface and alleviated the accumulation of plastic deformation in the soil but also enhanced the overall load-bearing synergy, highlighting notable structural advantages under high loads and complex service conditions.

3. Service life predictions based on the time-dependent diffusion coefficient-modified Fick’s second law revealed that the ternary blend concrete (KMS33) achieved a service life of up to 31.5 years, extending by 7.4 years and 13.3 years compared with slag-only and slag–fly ash concretes, respectively. These findings demonstrate that the synergistic effect of material proportion optimization and structural geometric improvement can effectively delay chloride-induced deterioration, thereby significantly enhancing the durability and reliability of marine piles. This provides a solid theoretical foundation and practical pathway for the long-service-life design of pile foundations in coastal engineering.

## Figures and Tables

**Table 1:** Chemical composition of powders.

Material	Chemical Composition (%)									
	SiO_2_	CaO	MgO	Fe_2_O_3_	Al_2_O_3_	SO_3_	K_2_O	Na_2_O	Alkali Content	Loss on Ignition
P·O 52.5	21.55	60.58	1.23	3.12	5.85	2.55	0.45	0.16	0.48	4.65
Fly Ash	55.94	1.53	0.68	3.16	33.38	0.17	0.69	0.46	/	/
Ground Slag	35.40	41.19	3.76	1.37	13.18	1.65	0.59	0.20	0.59	/

**Table 2:** Physical properties of aggregates.

Materials	Fineness Modulus	Apparent Density (kg/m^3^)	Dry Density (kg/m^3^)	Dry Water Absorption (%)	Flaky Content (%)	Clay Content (%)	Crushing Index (%)
River Sand	2.8	2640	2610	0.7	/	/	/
Crushed Stone	/	2660	2630	0.75	2	0.22	8.5

**Table 3:** Performance test results of admixtures.

Materials	Water Reduction Rate (%)	Water Bleeding Ratio (%)	Shrinkage Ratio (%)	Air Content (%)	Time Difference for Setting (min)		Compressive Strength Ratio (%)		
					Initial Set	Final Set	3 d	7 d	28 d
Fat-based Water-Reducing Agent	18.0	10	112	2.0	+30	−10	145	138	131
GB8076	≥14	≤90	≤135	≤3.0	−90~+120		≥130	≥125	≥120
Air-Entraining Agent HK-F2	6.5	58	124	4.6	+25	+10	96	97	91
GB8076	≥6	≤70	≤135	≥3.0	−90~+120		≥95	≥95	≥90

**Table 4:** Quality specifications of corrosion inhibitor.

Materials	Fineness (Residual on 45 μm Sieve) (%)	Moisture Content (%)	Loss on Ignition (%)	28-Day Activity Index (%)
Corrosion Inhibitor	≤12	≤1.0	≤8.0	≥75

**Table 5:** Corrosion-resistant concrete mix design.

Specimen Number	Fly Ash Content (%)	Slag Content (%)	Corrosion Inhibitor Content (%)	Raw Material Dosage per Cubic Meter of Concrete (kg/m^3^)								Water-Reducing Agent (%)	Air-Entraining Agent (%)
				Binder Content	Cement	Fly Ash	Slag	Corrosion Inhibitor	Sand	Small Gravel	Water		
K33	0	40	0	448	269	0	179	0	621	1200	148	1.2	0.1
KM33	10	30	0	442	265	44	133	0	621	1200	146	1.2	0.1
KMS33	10	22	8	433	260	43	95	35	639	1188	143	1.8	0.2

**Table 6:** Material physical properties’ parameters.

Materials	Elastic Modulus (MPa)	Density (kg·m^−3^)	Poisson’s Ratio	Internal Friction Angle (°)	Cohesion (kPa)	Expansion Angle (°)
Excavation Soil	23.5	1870	0.4	8.8	13.4	2.6
Concrete	37,500	2700	0.3	/	/	/

**Table 7:** Service life prediction of reinforced concrete considering chloride ion diffusion.

Specimen Number	Chloride Ion Diffusion Coefficient (×10^−12^ m^2^/s)	Service Life Prediction (Years)
K33	1.37	24.1
KM33	1.77	18.2
KMS33	1.14	31.5

## Data Availability

The original contributions presented in this study are included in the article. Further inquiries can be directed to the corresponding authors.
